# Complications of Total Laparoscopic Hysterectomy in a Tertiary Care Center

**DOI:** 10.7759/cureus.36510

**Published:** 2023-03-22

**Authors:** Benudhar Pande, Pranati Pradhan, Subrat K Pradhan, Sumitra Mansingh, Sanjeeb K Mishra

**Affiliations:** 1 Obstetrics and Gynaecology, Veer Surendra Sai Institute of Medical Sciences and Research, Sambalpur, IND; 2 Community Medicine, Veer Surendra Sai Institute of Medical Sciences and Research, Sambalpur, IND; 3 Field Epidemiology, Indian Council of Medical Research, Chennai, IND

**Keywords:** safe surgery, surgical complication, laparoscopic gynecology, minimally invasive laparoscopy, total laparoscopic hysterectomy

## Abstract

Introduction

Laparoscopic hysterectomy is a standard practice in developed countries and corporate setups in India but is a relatively new practice in government institutions; surgical audits are rarely done in our institutions. This study aims to determine the complications of total laparoscopic hysterectomy (TLH) in a tertiary care center in India.

Methods

This was a retrospective record review of patients admitted to the Obstetrics and Gynecology department of Veer Surendra Sai Institute of Medical Sciences and Research (VIMSAR), Odisha, India. Data were collected from case sheets of patients who underwent TLH, operated on between January 2018 and May 2022. Demographic and clinical data were extracted and analyzed.

Results

Of the 223 consecutive patients, 12 (5.3%) were converted to laparotomy. The mean age of patients was 44.34 years (±5.457), with a mean BMI of 24.24 kg/m^2^ (±2.181). The mean surgical duration was 1.895 hr (±0.487), with a mean blood loss of 140 ml and an average hospital stay of 3.25 (±0.821) days. Duration of surgery, blood loss, and hospital stay decreased with the surgeon's increasing experience. Reoperation was not needed in any case. Complications were recorded in about 18 (8.07%) cases. Also, major complications were less.

Conclusion

Total laparoscopic hysterectomy is a less invasive and safe alternative to total abdominal hysterectomy, offering the benefits of minimally invasive surgery, and is also ideal for candidates unsuitable for vaginal hysterectomy.

## Introduction

Hysterectomy is the most common gynecological operation performed on women. Laparoscopy avoids the need for laparotomy in most cases. Total laparoscopic hysterectomy (TLH), i.e., type VII, is a laparoscopic hysterectomy in which the entire surgical procedure, including the vault closure, is carried out via a laparoscope [1]. After detachment, the specimen is removed vaginally or by tissue extraction technique. The advantages of TLH over abdominal hysterectomy (AH) and laparoscopically assisted vaginal hysterectomy (LAVH) are rapid recovery, short hospitalization, less blood loss, and fewer minor complications of the wound or abdominal wall [2]. Limiting factors include a longer operating time, skilled hands, and a higher incidence of urinary tract injuries [3]. Vaginal hysterectomy (VH) and LAVH are suitable for at least moderate prolapse patients. In the case of nulliparous patients, vaginal dissection is difficult due to the lack of prolapse and relatively small vaginal capacity, resulting in higher complications [4].

Different studies have shown that the complication rate of total laparoscopic hysterectomy is up to 10% [5,6]. TLH requires a greater degree of surgical competence and a more elaborate learning curve [7]. A newly trained gynecologist must become proficient and confident enough to perform it. If TLH method safety were confirmed in a sizable number of retrospective series, it would attract more surgeons to learn and safely perform laparoscopy in contrast to open techniques.

Very few government hospitals and institutions in the state have successfully implemented this advanced mode of surgery in their day-to-day practice. Excess patient load, fear of complications, and costly equipment maintenance prevent surgeons from using them in a government hospital, and laparoscopic surgeries are primarily performed in a corporate setup. Our study reviewed the data for minimal access surgery as a treatment modality for various gynecological conditions. We have evaluated the advantages and safety of TLH, mainly within the constraints of a government setup, with the objective of describing the complications of total laparoscopic hysterectomy in a tertiary care setup in India.

## Materials and methods

This study is a retrospective record review. All TLH cases were identified from the laparoscopy operation theatre register. Bed head tickets were reviewed for details about the cases. Data for TLH performed in every benign gynecologic indication like fibroid, adenomyosis, prolapse, abnormal uterine bleeding (AUB), etc., Stage Ia1 cervix cancer, ovarian cancer (occult), and Stage IIA or less endometrial cancer were collected. Surgery was performed by senior faculties of the Obstetrics and Gynaecology (O&G) department from January 2018 to May 2022 at the Veer Surendra Sai Institute of Medical Sciences and Research (VIMSAR, Sambalpur) O&G department, assisted by an assistant professor or resident. Ethical approval was obtained from Institutional Research and Ethics Committee.

The technique used for TLH in the hospital is described in detail here. The patient's position was modified to lithotomy with hips extended at a 40-degree angle in the Trendelenburg position in booted support stirrups. The sandbag was placed below the buttock. The arms were cushioned and tucked by the side, while shoulder bolsters prevented slipping up the table. Then bimanual inspection was completed. After the examination, the patient was anesthetized, and a Mangeshikar-style uterine manipulator with a cupping device was inserted. It helped in delineating the cervicovaginal junction and for colpotomy and final tissue extraction. The manipulator was introduced in cases of endometrial cancer following laparoscopic washings and bipolar cautery blockage of the proximal fallopian tubes. A Foley catheter was given. The position of the monitor was adjusted. A safety trocar was inserted above the umbilicus by open technique. Lateral ports were inserted at the lateral margin of the rectus muscle three to four finger breadths medial to anterior superior iliac spine under transillumination. The second lateral port was inserted four fingers above and medial to the first lateral port. Similarly, two lateral ports were given on the opposite side.

A thorough abdomino-pelvic survey was done, and ureters were identified. The round ligament was grasped, coagulated and incised. In case of ovaries' removal, the infundibulopelvic (IP) ligament was pulled up and away from retroperitoneal structures, and was coagulated and incised with a shearer or bipolar and scissors. The proximal portions of the fallopian tube and utero-ovarian ligament are desiccated and transected when ovaries are conserved. The uterus, on the uterine manipulator, was pushed cephalad to create the "traction counter-traction" concept of open surgical dissection of the lower uterine segment. This elevates the uterine arteries along the lower cervix away from the ureter. A bladder flap was incised, and the anterior cervical fascia was exposed. This dissection was done with a shearer or with a bipolar cautery. The uterine arteries were coagulated with the bipolar and incised, and thus pushed downward and laterally to expose the cardinal ligament fibers. The cardinal ligament were incised posteriorly to the uterosacral ligaments and inferiorly, identifying the cervicovaginal margin as the lowest limit of dissection.

The cervicovaginal margin is laparoscopically "palpated" with the laparoscopic instruments using visual and manual clues to delineate the posterior margin, the lateral edges, and the anterior margin of the cervical stroma. Typically, the cervical stroma is firm and moves as a solid object, and the vaginal wall is pliant and dimples with pressure. The vagina is incised at the precise margin of the cervix and vagina by a shearer or monopolar cautery. A tenaculum is inserted through the vagina to grasp the cervix and remove the uterus. When the pneumoperitoneum is lost, the uterine manipulator is removed, and a surgical glove containing fluffed gauze pieces is inserted to obliterate the lower vagina.

When the uterus is large, it is morcellated transvaginally. The vaginal apex is closed with a vicryl suture with graspers and a needle holder, fixing the vaginal angle to the uterosacral ligaments for suspension. Standard oncological principles were followed in all malignancy cases; pelvic washings were done and pelvic masses were removed intact in an endo bag by morcellation; uteri were removed without morcellation or in a bag. Lymph node dissections were done when indicated. Surgical procedures were never compromised by minimally invasive techniques. Descriptive statistics were generated related to sample characteristics and other variables of interest.

## Results

Of 223 patients recorded eligible for this study, 12 (5.3%) were converted to open laparotomy. Four had multiple leiomyomas. In one case, myoma was in the broad ligament, and in the other three, in the lower uterine segment obstructing laparoscopic ligation of the uterine arteries. One patient was converted due to bladder injury, which was a problematic case of three previous lower segment cesarean sections (LSCSs). Two cases were converted because of adhesions: a case of endometriosis and a patient of previous LSCS. The rest of the patients were converted due to hemorrhage. Therefore, about 5.38% of all laparoscopic hysterectomy procedures failed and needed laparotomy.

The mean age of the patients was 44.34 years (±5.46). The average parity was two. The nulliparous rate among these women was about 6.4%. Their average BMI was 24.24 kg/m^2^ (±2.18). Leiomyoma uteri was the most common preoperative diagnosis followed by abnormal uterine bleeding (Table 1).

**Table 1:** Preoperative diagnosis of cases of total laparoscopic hysterectomy AUB, abnormal uterine bleeding; L, Lateral; P, posterior; A, anterior; HSIL, high-grade squamous intraepithelial lesion; LSIL, low-grade squamous intraepithelial lesion; COEIN, coagulopathy, ovulatory dysfunction, endometrial, iatrogenic, not yet classified

Preoperative diagnoses	Number of cases (%)
Leiomyomata uteri	38 (17.0%)
AUB (COEIN)	71 (31.8%)
AUB-L	37 (16.5%)
AUB-A	11 (4.4%)
AUB-P	5 (2.2%)
Endometrial hyperplasia	13 (5.8%)
Endometrial carcinoma	2 (0.8%)
Endometritis	1 (0.4%)
Prolapse with stress incontinence	1 (0.4%)
Leiomyoma with adenomyosis	2 (0.8%)
Adenomyosis	25 (11.2%)
Endometriosis	4 (1.7%)
Cervical dysplasia	1 (0.4%)
HSIL	3 (1.3%)
LSIL	2 (0.8%)
Cervical carcinoma	1 (0.4%)
Hydrosalpinx	1 (0.4%)
Ovarian cyst	2 (0.4%)
Chocolate cyst of ovary	1 (0.4%)
Ovarian carcinoma	2 (0.8%)

Table 2 lists other procedures carried out in conjunction with total laparoscopic hysterectomy. The length of the surgery, which comprised all gynecologic procedures, was extrapolated from operating room records. The duration analysis did not include patients who required an appendectomy, cholecystectomy, or other general surgical operations such as staging lymphadenectomy and omentectomy for cancer. The mean surgery duration was 1.895 hr (±0.4876; range, 1-3). Surgery on more than a fifth (23.7%) of patients was completed within 60 min or less. Age, BMI, or parity did not affect the length, but the surgeon's increased experience over time cut down on time.

**Table 2:** Additional procedures performed with total laparoscopic hysterectomy

Procedure	Number (%)
Bilateral salpingo-oophorectomy	133 (59.6%)
Salpingo-oophorectomy	2 (0.89%)
Appendectomy	1 (0.44%)
Lymph node dissection	2 (0.89%)
Uterosacral ligament plication	1 (0.44%)
Excision of endometriosis	4 (1.79%)
Ureterolysis	2 (0.89%)
Omentectomy	1 (0.44%)
Posterior repair	1 (0.44%)
Sacropexy	1 (0.44%)

Blood loss was approximated by looking at the canister's contents before irrigation or by agreement between the surgeon and the anesthesiologist. The average blood loss was 140 ml, with 10% of patients losing less than an estimated 10 ml of blood. This estimate is made when there is almost little blood loss seen throughout the surgery, little to no blood loss from the laparoscopic incisions, and little to no blood loss seen in the pelvic cul de sac after the conclusion of the treatment. More than 50% of the patients lost less than 50 ml blood. The mean weight of the uterus was 120 gm, while the median weight was 180 gm. Three percent of women needed transfusions, primarily given in hemorrhagic complications. Reoperation was not required in any of the cases. However, those requiring transfusions had more extended surgery and more days in the hospital. The average hospital stay was 3.21 days (±0.82). Complications occurred in 18 patients. Bladder injury occurred in a case with a history of three previous cesarean sections. Vaginal vault infections occurred in five patients, out of which postoperative vaginal vault bleeding occurred in four patients (1.7%). Port site pain and infection were there in one case. Hemorrhage occurred in 12 cases, among which eight were converted to TAH with bilateral salpingo-oophorectomy (BSO). Most cases were of multiple fibroids with fibroids in a lower segment or cervical fibroids. There was no bowel or ureteric injury, postoperative pelvic bleed, or retroperitoneal hematoma. Urologic complications occurred in one (0.4%) patient. No reoperations were needed. Excluding the cases converted to laparotomy, major complications occurred in 0.4% of completed TLH. Complications during TLH are given in Table 3.

**Table 3:** List of complications during total laparoscopic hysterectomy

Complications	Frequency (%)
Hemorrhage	12 (5.3%)
Bladder injury with repair	1 (0.44%)
Ureter injury	0
Intestinal injury	0
Vaginal cuff bleeding	4 (1.7%)
Vaginal cuff Infection	1 (0.44%)
Trocar site infection	1 (0.44%)

## Discussion

Total laparoscopic hysterectomy is being increasingly adopted by gynecologists as a type of minimally invasive surgery because of its many advantages over open surgery, such as less complications like minimal blood loss, decreased hospital stay, less pain, early recovery, etc. Unlike vaginal hysterectomy, total laparoscopic hysterectomy is independent of the vaginal size or capacity.

In this study, we aimed to find out the complications of TLH irrespective of sociodemographics or clinical presentation. Leiomyomata uteri, abnormal uterine bleeding, endometrial hyperplasia and adenomyosis were the major indications for total laparoscopic hysterectomy. Ashfaq et al. reported that the most common indication for TLH was uterine fibroids (54% ) and AUB (36%) [5]. Bilateral salpingo-oophorectomy was done as an additional procedure in more than half of the patients. A total of 12 patients were converted to open laparotomy to manage the complications and which were not operable by the total laparoscopic method. Patients with a greater risk of laparotomy may be identified by preoperative patient evaluation and fibroid mapping in lower uterine segment fibroids.

In this study, the total complication rate was 8.07%, and the major complication rate was less than 1%. The overall and major complication rates reported by Hoffman et al. were 10% and 5.6%, respectively [8]. Heinberg et al. reported a major complication rate of 14.4%, and Chapron highlighted that of those who underwent total laparoscopic hysterectomy, 10% experienced complications [9,10]. Many researchers reported the major and minor complications separately. Total laparoscopic hysterectomy has been shown to be valuable, as observed from surgical data in most cases.

Hemorrhage occurred in 12 (5.3%) patients and because of this complication, eight patients were converted to other modalities of operative management. Similar findings on hemorrhage were also reported by different authors [6,11]. The risk of hemorrhage also increases with the increase in the uterus size as observed by Bonilla et al. [12]. Three percent of women needed transfusions, primarily given in hemorrhagic complications. Type VII total laparoscopic hysterectomy demonstrated decreased rates of complications, less postoperative pain and discomfort, less blood loss, and significant financial savings due to lower hospital expenses. However, we recommend to measure blood loss more precisely.

Only one patient was subjected to bladder injury and the bladder was repaired immediately. Bladder injury occurred in a patient with a history of three previous cesarean sections. The repair was successful and no further clinical complaint was made by the patient during the hospital stay. Cystoscopy after total abdominal hysterectomy was performed in the patient to confirm the closure of the repaired defect. In a study, no bladder injury was reported; however, the number of patients who underwent total laparoscopic hysterectomy was very less [5]. Urologic injuries reported by O’Hanlan et al. were less than 3% [6].

Vaginal cuff infection occurred in one patient and one patient developed trocar site infection. Both the patients were treated with antibiotics and recovered during hospital stay. A similar finding was reported by another author [13]. Vaginal cuff bleeding occurred in 1.7% patients, which is higher than the complication rate reported by Mereu et al. [14]. The average hospital stay was 3.21 days (±0.82). The average hospital stay was 2.06 (±0.3) days in a study by Roman [15]. In comparison to the hospital stay for open surgery, the hospital stay was shorter that resulted in early returning to normal activities by the patient. A shorter hospital stay also reduces the out-of-pocket expenditure by the patients and their families.

As a medical record review, the study has many limitations. All the patients were operated in a single center only. Also, the period of follow-up of patients was very less. The quality of data abstracted from the record is prone to bias. In addition, we did not investigate other information that may have influenced the occurrence of complications.

## Conclusions

In patients with a pelvic mass, endometriosis, adhesions, or cancer, total laparoscopic hysterectomy is relatively risk-free and provides good access to the whole abdomen as needed with little morbidity. It is beneficial in obese, nulliparous, and diabetic women. With a proper selection of cases and an experienced hand, complications from this technique will be lesser. Also, an appropriate understanding of laparoscopic surgical techniques will defeat the difficulties encountered during the procedure. Surgeons can safely learn TLH and help in reducing its limitations, such as longer operating time. Hence, TLH should be an integral part of the basic knowledge of a gynecological surgeon.
